# Stability Assessment of Compounded Niaprazine Oral Solutions to Support an Evidence-Based Beyond-Use Date

**DOI:** 10.3390/pharmaceutics18081006

**Published:** 2026-08-14

**Authors:** Antonio Lopalco, Borja Martínez-Alonso, Marina Cortellino, Cosimo Annese, Alexia Barbarossa, Catiana Mirgaldi, Angela Sanrocco, Stefania Antonacci, Sergio Fontana, Angela Assunta Lopedota, Nunzio Denora

**Affiliations:** 1 Department of Pharmacy-Pharmaceutical Sciences, University of Bari Aldo Moro, 70125 Bari, Italy; m.cortellino5@phd.uniba.it (M.C.); alexia.barbarossa@uniba.it (A.B.); angelaassunta.lopedota@uniba.it (A.A.L.); 2 Department of Biomedical Sciences, Faculty of Pharmacy, University of Alcalá, Alcalá de Henares, 28871 Madrid, Spain; borja.martineza@uah.es; 3 Centro Studi e Ricerche “Dr. S. Fontana 1900–1982”, Farmalabor s.r.l., 47, Piano S. Giovanni Street, 76012 Canosa di Puglia, Italy; s.fontana@farmalabor.it; 4 Department of Life Sciences, Health, and Health Professions, Università degli Studi Link, Via del Casale di San Pio V, 00165 Roma, Italy; c.annese@unilink.it; 5 Department of Chemistry, Università degli Studi di Bari Aldo Moro, Via Orabona 4, 70126 Bari, Italy; 6 Dipartimento Gestione del Farmaco—Azienda Sanitaria Locale Bari, Lungomare Starita 6, 70123 Bari, Italy; mirgaldicatiana@gmail.com (C.M.); angela.sanrocco@asl.bari.it (A.S.); stefania.antonacci@asl.bari.it (S.A.)

**Keywords:** niaprazine, extemporaneous, pediatric, oral, compounding, formulation, stability, LC-MS, HPLC-DAD, microbiological

## Abstract

**Background/Objectives**: Niaprazine is widely used for the management of sleep disorders in pediatric and geriatric patients; however, no commercially available oral liquid formulation is currently available in Italy, making extemporaneous compounding necessary. In routine practice, the beyond-use date (BUD) is often limited to 30 days, potentially affecting therapeutic continuity. This study aimed to evaluate the chemical, physical, and microbiological stability of a compounded niaprazine syrup to support evidence-based BUD and to assess the suitability of selected ready-to-use compounding vehicles for preparing alternative niaprazine oral liquid formulations. **Methods**: A niaprazine syrup (3 mg·mL^−1^) was prepared in a sucrose-based vehicle acidified with tartaric acid and preserved with potassium sorbate. Chemical stability of niaprazine was evaluated by high-performance liquid chromatography coupled with diode array detector (HPLC-DAD), whose specificity was confirmed by forced degradation studies. Stability was monitored for up to 9 months at 4–8, 25, and 40 °C and confirmed after 12 months by HPLC-DAD and mass spectrometry (MS). Physical stability of the formulation was monitored by pH and visual inspection up to 12 months. Microbiological quality was assessed for 2 months at 4–8 and 25 °C according to the European Pharmacopoeia. In parallel, four selected ready-to-use compounding vehicles were evaluated for their suitability to prepare stable niaprazine oral liquid formulations. **Results**: Niaprazine concentrations in the syrup remained within pharmacopeial acceptance limits (±10%) at all temperatures, although a decrease was observed under accelerated conditions (40 °C). pH remained stable (≤0.5-unit variation) and the formulation stayed clear and homogeneous throughout the study, with only minor visual changes after prolonged storage at 40 °C. Statistically significant differences (*p* < 0.05) were observed in both the HPLC-DAD and HPLC-MS datasets. Microbiological testing confirmed compliance up to 2 months at 4–8 and 25 °C (TAMC ≤ 10^3^ CFU/mL; TYMC ≤ 10^2^ CFU/mL; *Escherichia coli* absent). Comparable chemical and physical stability was observed for three of the four formulations prepared with the ready-to-use vehicles over at least two months. **Conclusions**: The compounded niaprazine syrup demonstrated chemical, physical, and microbiological stability under refrigerated and room-temperature storage, supporting evidence-based beyond-use dating of up to two months under the tested conditions. Ready-to-use vehicles may represent a practical complementary approach, offering standardized alternatives for the preparation of niaprazine oral liquid formulations.

## 1. Introduction

**Figure 1:**
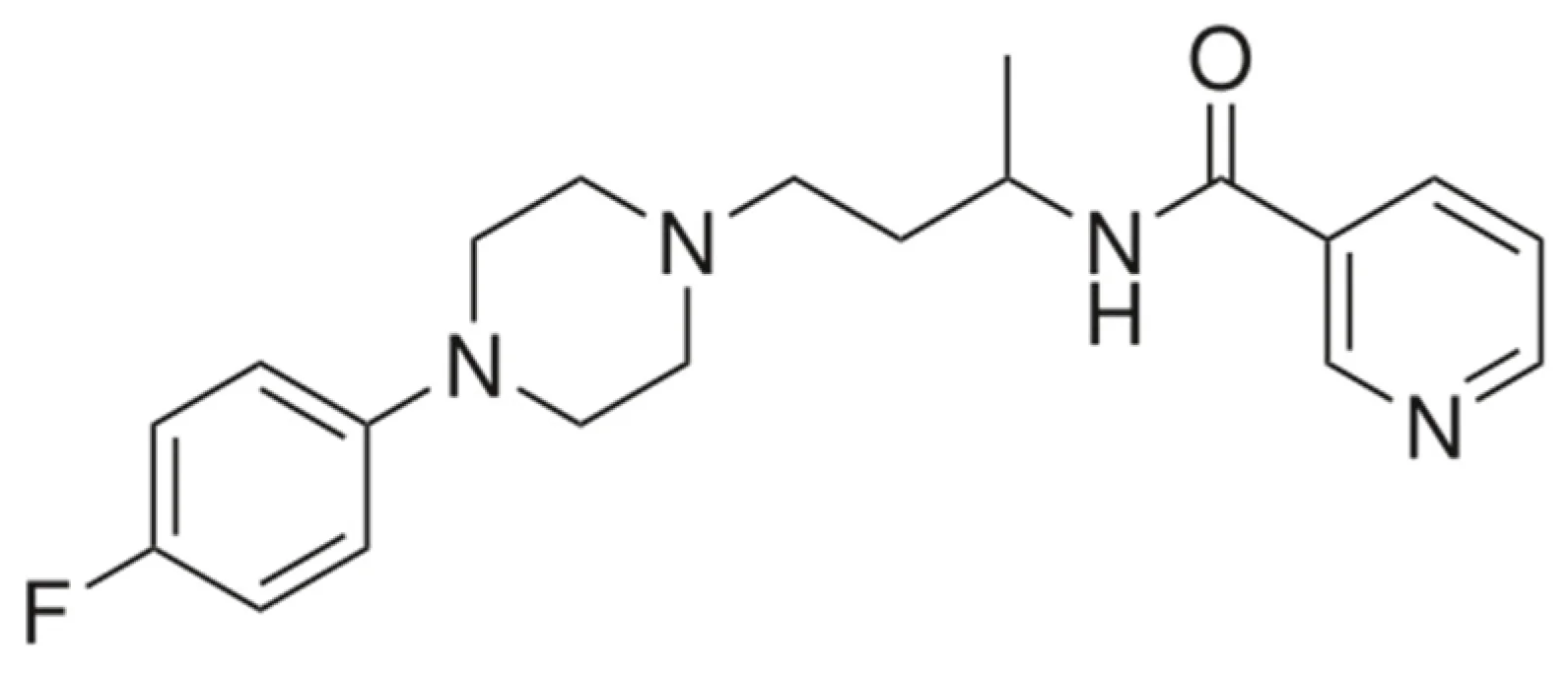
Chemical structure of niaprazine.

Niaprazine (N-[3-[4-(p-fluorophenyl)-1-piperazinyl]-1-methylpropyl]-nicotinamide) is a phenylpiperazine derivative initially described as a first-generation antihistamine (Figure 1). Early pharmacological characterization attributed antihistaminic and anticholinergic properties to this compound. However, subsequent studies demonstrated negligible affinity for histamine H_1_ and muscarinic cholinergic receptors, highlighting instead selective interactions with specific serotonergic and α-adrenergic receptor subtypes [1]. This receptor binding underlies a pharmacodynamic behavior distinct from that of classical sedating antihistamines, with predominant modulation of serotonergic pathways involved in sleep regulation, particularly those associated with slow-wave sleep, while relatively sparing noradrenergic circuits implicated in rapid eye movement (REM) sleep [2].

Niaprazine is a weakly basic piperazine derivative (pKa ≈ 7.9) that is practically insoluble in water under neutral conditions (0.171 mg/mL). Its aqueous solubility markedly increases under mildly acidic conditions owing to protonation of the piperazine moiety, making pH control a critical factor in formulation development. Consequently, acidified aqueous vehicles are particularly suitable for maintaining complete drug dissolution, minimizing the risk of precipitation during storage, and ensuring the physicochemical stability and dose uniformity of compounded oral liquid formulations [3]. Owing to these properties, niaprazine has been used mainly for the management of sleep disorders characterized by difficulties in sleep onset and frequent nocturnal awakenings, especially in pediatric and geriatric patients [4]. In adults, the recommended dose ranges from 30 to 60 mg administered once daily before bedtime, whereas in pediatric patients the dosage was individualized according to body weight and clinical response, generally ranging from 1 mg/kg/day up to a maximum daily dose of 30 mg [5]. In geriatric patients, lower initial doses are generally recommended, with subsequent titration according to tolerability and therapeutic response. In Italy, niaprazine has been marketed as the industrial medicinal product *Nopron^®^* syrup (30 mg/10 mL) and coated tablets (30 mg); however, the products were withdrawn on 9 November 2012 following the marketing authorization holder’s decision not to renew the authorization [6]. Since then, its clinical use has relied exclusively on extemporaneously compounded oral formulations, prepared and dispensed under personalized medical prescription in accordance with Italian Law No. 94/1998 (the so-called “Di Bella Law”) [7,8]. According to compounding practice guidelines and pharmacopeial recommendations, aqueous oral liquid preparations lacking antimicrobial preservatives or containing less than 25% (*v*/*v*) ethanol are generally assigned a maximum beyond-use date (BUD) of 30 days [9,10]. This limitation represents a practical challenge in clinical practice, as it necessitates frequent preparation renewals and may negatively affect therapeutic continuity, healthcare costs, and patient adherence. In this context, ensuring the quality of compounded oral solutions requires a comprehensive assessment of both chemical and physical stability. Chemical stability refers to the ability of the active pharmaceutical ingredient (API) to maintain its chemical integrity and concentration within predefined limits over time, whereas physical stability of a formulation encompasses parameters such as appearance, color, odor, clarity, pH, and the absence of precipitation or phase separation. All these parameters directly influence the acceptability, quality, safety, and efficacy of a preparation [11,12]. Robust analytical methodologies are therefore essential to support evidence-based decisions regarding the BUD of magistral formulations. High-performance liquid chromatography (HPLC) coupled with a photodiode array detector (DAD) represents the reference technique for the quantitative evaluation of API content in stability studies. However, reliance on a single analytical approach may be insufficient to fully support conclusions on chemical stability. The use of orthogonal analytical techniques, such as liquid chromatography–mass spectrometry (LC–MS), is increasingly recommended to corroborate chromatographic data and to confirm the chemical integrity of the API over time [13,14]. In this context, LC–MS does not necessarily serve for the identification of degradation products, but rather as a complementary tool to HPLC, providing additional confidence in the assessment of chemical stability through molecular-level confirmation of the intact drug substance. Based on these considerations, the aim of the present study was to evaluate the chemical and physical stability of a compounded oral syrup of niaprazine, with the objective of assessing the feasibility of extending its BUD beyond the conventional 30-day limit applied to aqueous oral preparations. In addition, a further experimental phase was designed to investigate niaprazine behavior in four commercial ready-to-use vehicles. The evaluation of these vehicles was undertaken to provide a simpler approach to extemporaneous compounding, ensuring method standardization and reproducibility, while potentially offering additional advantages related to their optimized composition. Niaprazine 3 mg·mL^−1^ formulations were prepared and aliquoted into amber glass bottles. Samples were stored under controlled temperature conditions (4–8, 25 and 40 °C). Chemical stability of the API in all the formulations was assessed over time using a validated stability-indicating HPLC-DAD method supported by an LC-MS technique, while physical stability of the preparations was evaluated through systematic visual inspection and monitoring of their pH values. In addition, microbiological stability studies were conducted on the syrup formulation stored at 4–8 and 25 °C over a two-month period.

## 2. Materials and Methods

### 2.1. Materials

Pharmaceutical-grade niaprazine (Lot No. 0210623) was used as the API for the preparation of the formulations; potassium sorbate (Lot No. R2334053), tartaric acid (Lot No. R2323571), sucrose ph.eur. (Lot No. W2206296) were supplied by Farmalabor Srl (Canosa di Puglia, Italy) and purified water (Lot No. V5F554125F, Carlo Erba, Cornaredo, Italy). Fast oral solution Puccini (Lot No. W2404972), Mozart sugar free (Lot No. W2306324), Wagner (Lot No. W2402350) and Chopin (Lot No. G2400174) vehicles were supplied by Farmalabor Srl. Formic acid (≥95%, Lot No. P1540, Honeywell Fluka™, Seelze, Germany), sodium hydroxide (≥98%, Lot No. L137A, Honeywell Fluka™), hydrochloric acid (ACS reagent ≥ 37%, Lot No. L1060, Honeywell Fluka™), purified water for HPLC (gradient grade ≥ 99.9%, Lot No. M0780, Fisher Chemicals™, Waltham, MA, USA), methanol (HPLC gradient grade ≥ 99.9%, Lot No. P0550, Honeywell Riedel–de Haën™), and PERDROGEN™ hydrogen peroxide 30% (ISO grade ≥ 30%, Lot No. K1190, Honeywell Fluka™) were purchased from Lenvachimica S.r.l. (Bari, Italy). Volumetric flasks, vials, beakers, pipettes, and graduated cylinders, all made of borosilicate glass, were also obtained from Lenvachimica S.r.l. (Bari, Italy).

### 2.2. Preparation of Niaprazine Syrup Formulation

The composition of the niaprazine 3 mg·mL^−1^ syrup prepared by the Clinical Galenic Laboratory of the Territorial Pharmacy of Altamura (Bari, Italy) is reported in Table 1.

The formulation was prepared in accordance with the *Norme di Buona Preparazione* (NBP) of the Italian Official Pharmacopoeia, following the standardized operating procedures routinely adopted by the Clinical Galenic Laboratory for the compounding of non-sterile oral liquid preparations [15,16]. The required quantities of niaprazine and excipients were accurately weighed (Table 1). Simple syrup was prepared according to the Italian Pharmacopoeia [17]. In a beaker of adequate capacity, a sufficient amount of purified water (approximately 60 mL) was brought to boil to allow complete dissolution of the sucrose contained in the simple syrup. After obtaining a clear and colorless solution, the mixture was allowed to cool, and the antimicrobial preservative potassium sorbate (0.20% *w*/*v*) was added under stirring. Niaprazine and tartaric acid were separately dissolved in purified water and subsequently added to the sucrose solution under continuous agitation. The medicated syrup was finally brought to final volume of 150 mL with purified water. The final pH value of the formulation was approximately 5.0, a range compatible with both complete drug dissolution and potassium sorbate preservative activity. The final niaprazine 3 mg·mL^−1^ formulation was divided into six pharmaceutical-grade amber glass bottles (30 mL nominal capacity), each containing 25 mL of syrup. The bottles and screw-cap closures were certified as primary packaging materials for non-sterile oral liquid preparations, compliant with the requirements of the Italian Official Pharmacopoeia, and supplied by Farmalabor s.r.l. The containers were tightly sealed immediately after filling and remained closed throughout the storage study, except during scheduled sampling.

### 2.3. Preparation of Niaprazine Formulations in Ready-to-Use Bases and Stability Studies

A preliminary qualitative assessment of the ability of four commercially available ready-to-use oral liquid bases (Table 2) to dissolve niaprazine at the target concentration of 3 mg·mL^−1^ was performed at room temperature. Following drug addition under continuous stirring, each formulation was visually inspected for clarity, homogeneity, and the absence of undissolved particles, precipitation, or phase separation. Based on this preliminary assessment, three of the four tested bases (Puccini, Mozart sugar free, and Wagner) were selected for further investigation, as they produced clear and homogeneous formulations at the desired concentration. Niaprazine formulations (3 mg·mL^−1^) were prepared in the selected vehicles by dissolving the accurately weighed API under continuous stirring and bringing the formulation to the final volume with the corresponding vehicle. The formulations were then aliquoted into appropriate amber containers and used for further studies.

### 2.4. High-Performance Liquid Chromatography (HPLC) Coupled with Photodiode Array Detection (HPLC–DAD) and Analytical Conditions

The HPLC analysis was conducted following the method described by Trebesova et al. [18]. A Shimadzu Nexera series HPLC equipped with a DAD and a SIL-40C autosampler was used [19] (software: LabSolutions DB/CS, Shimadzu Corporation, Kyoto, Japan). An Eclipse Plus C18, 4 µm, 4.6 × 250 mm, with a guard pre-column (Agilent Technologies, Santa Clara, CA, USA) was used. The mobile phase was composed of 35% *v*/*v* methanol and 65% *v*/*v* water (pH~2.7), containing 0.1% *v*/*v* of formic acid. The flow rate and the temperature were set at 0.8 mL·min^−1^ and 30 °C, respectively, and ultraviolet (UV) detection was carried out at a wavelength (λ) equal to 245 nm. A calibration curve was constructed by solubilizing 5.0 mg of niaprazine with methanol in a 5 mL volumetric flask. Consecutive dilutions were obtained using the mobile phase, and the linearity of the HPLC method was demonstrated in a concentration range between 1000 μg·mL^−1^ and 1 μg·mL^−1^. Each analysis lasted 30 min and the volume of each injected sample was 10 μL. Each calibration level was injected in triplicate and the mean peak area was plotted against nominal concentration. Linearity was evaluated and demonstrated by least-squares linear regression of peak area versus concentration across the calibration range, and the coefficient of determination (R^2^ = 0.9998) was recorded. Limit of Detection (LoD) and Limit of Quantification (LoQ) were calculated from the calibration slope (S) and the instrument noise (σ) measured from six blank injections using Equations (1) and (2), respectively:

(1) LoD = 3.3 σ/S

(2) LoQ = 10 σ/S

The resulting values of LoD and LoQ were 1.37 × 10^−6^ mg·mL^−1^ (1.37 ppb) and 4.15 × 10^−6^ mg·mL^−1^ (4.15 ppb), respectively. All reported niaprazine concentrations were obtained by interpolation from the calibration curve.

### 2.5. Forced Degradation Studies

To confirm that the HPLC-DAD method was suitable for a stability study, niaprazine was subjected to forced-degradation tests (Table 3). This study was designed to determine whether potential degradation products interfered with the API peak [20]. Forced-degradation studies were performed on niaprazine formulations (3 mg·mL^−1^) and a 1 mg·mL^−1^ stock solution of drug dissolved in methanol. Samples were prepared in duplicate and forced degradation conditions included acidic, alkaline, peroxide, UV light and thermal exposure. For acidic degradation 0.8 mL of HCl 5N were added to a volumetric flask containing 0.2 mL of niaprazine methanol solution or 0.2 mL of each formulation. Similarly, caustic degradation was performed by mixing the sample (0.2 mL of stock solution or 0.2 mL of formulation) and 0.8 mL of NaOH 5N. For the oxidative degradation 0.8 mL of 30% *w*/*w* H_2_O_2_ were added to 0.2 mL of the tested samples. For UV exposure and thermal exposure, 0.1 mL of samples (niaprazine stock solution or formulation) were transferred to a volumetric flask, diluted with purified water and kept in a light-protected chamber, constantly irradiated by UV lamp (λ = 365 nm), or in a thermostatically controlled chamber at 70 °C. Stress tests were carried out for 72 h at 25 °C, after which the samples subjected to acid and alkaline degradation were neutralized with 0.8 mL of 5N NaOH and 0.8 mL of 5N HCl, respectively. Oxidative stress tests were conducted for 1 h at 25 °C. Subsequently, all samples were analyzed using the HPLC method described previously.

### 2.6. High-Performance Liquid Chromatography Coupled to Diode Array Detection and Mass Spectrometry (HPLC–DAD–MS)

#### 2.6.1. Instrumentation and Chromatographic Conditions

Chromatographic analyses of the samples stored for 12 months at the three different temperatures were performed using a Shimadzu LC/MS-ESI-IT-TOF system equipped with a DAD and an electrospray ionization (ESI) source coupled to an ion trap–time-of-flight (TOF) mass analyzer (Schimadzu HPLC Nexera series, equipped with SPD-M40 photodiode array detector, SIL-40C autosampler, and CTO-40C column oven, Shimadzu Corporation, Kyoto, Japan). Chromatographic separation was achieved on a GraceSmart RP-18 column (5 μm particle size, 150 mm × 4.6 mm i.d.). The mobile phase consisted of a mixture of water/methanol (65:35, *v*/*v*) containing 0.1% (*v*/*v*) formic acid, delivered under isocratic conditions at a flow rate of 0.8 mL·min^−1^, as described in Section 2.3. *High-performance liquid chromatography with diode array detection (HPLC–DAD) and analytical conditions*. The injection volume (10 μL) was kept constant for all analyses.

#### 2.6.2. Detection Conditions

Analyses were carried out using dual detection, combining UV and mass spectrometric (MS) detection. UV detection was performed using a DAD in the wavelength range of 200–600 nm, with quantitative measurements acquired at λ = 254 nm. Mass spectrometric detection was conducted in both positive and negative ionization modes, scanning a mass range of *m*/*z* 50–1000. Nitrogen was used as both nebulizing and drying gas. The nebulizing gas flow was set at 1.5 L/min, while the drying gas pressure was maintained at 102 MPa with a desolation temperature of 250 °C.

#### 2.6.3. Quantification Strategy and Internal Standard

**Figure 2:**
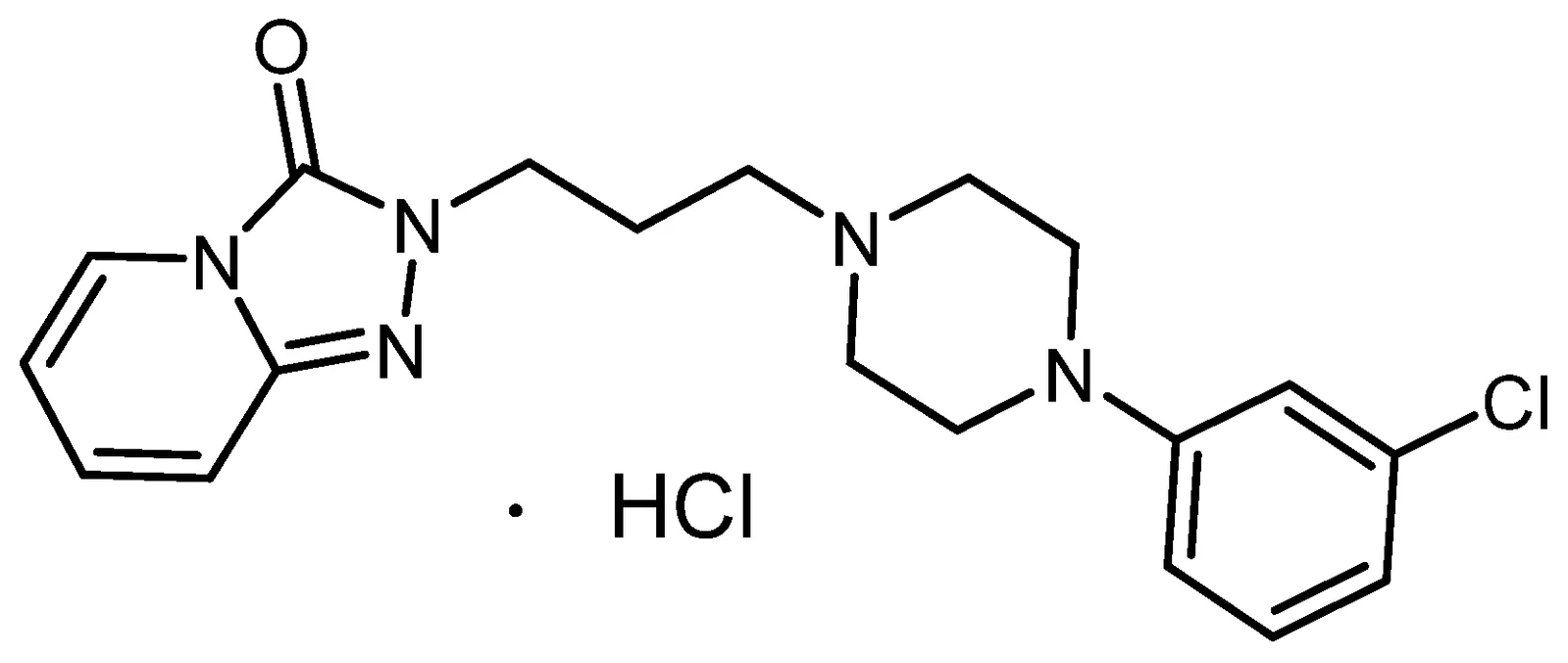
Trazodone hydrochloride chemical structure.

Niaprazine was quantified using both diode array and mass detection by means of calibration curves constructed according to the internal standard method. Trazodone hydrochloride (Figure 2) was selected as the internal standard (ISTD) due to its suitable chromatographic behavior and ionization properties under the selected analytical conditions. Quantification was based on the ratio between the analytical response of niaprazine and that of the ISTD, calculated for both UV absorbance at 254 nm and total ion current (TIC) signals.

#### 2.6.4. Preparation of Calibration Standards and Analysis

Calibration curves were constructed using five standard solutions, prepared by appropriate dilution of a niaprazine stock solution (0.900 mg·mL^−1^) and a trazodone hydrochloride stock solution (1.255 mg·mL^−1^). The mobile phase was used as the diluent for all solutions. Each calibration standard was prepared to a final volume of 10 mL, maintaining a constant ISTD concentration (0.0376 mg·mL^−1^) by adding 0.3 mL of trazodone hydrochloride stock solution, while varying the niaprazine concentration over the range 0.0045–0.090 mg·mL^−1^. The composition of the calibration standards is reported in Table 4.

Quantitative analysis was based on the following relationship (Equations (3) and (4)):

(3) As/AISTD = f × Cs/CISTD

where C_S_ and C_ISTD_ are the concentrations of niaprazine and the internal standard, respectively, A_S_ and A_ISTD_ are the corresponding instrumental responses, and f is the proportionality factor.

Since the ratio C_ISTD_/f remains constant, Equation (3) can be simplified as:

(4) As/AISTD = k × Cs

where k is a constant. Calibration curves were obtained by plotting the response ratio A_s_/A_ISTD_ versus the niaprazine concentration C_S_.

**Figure 3:**
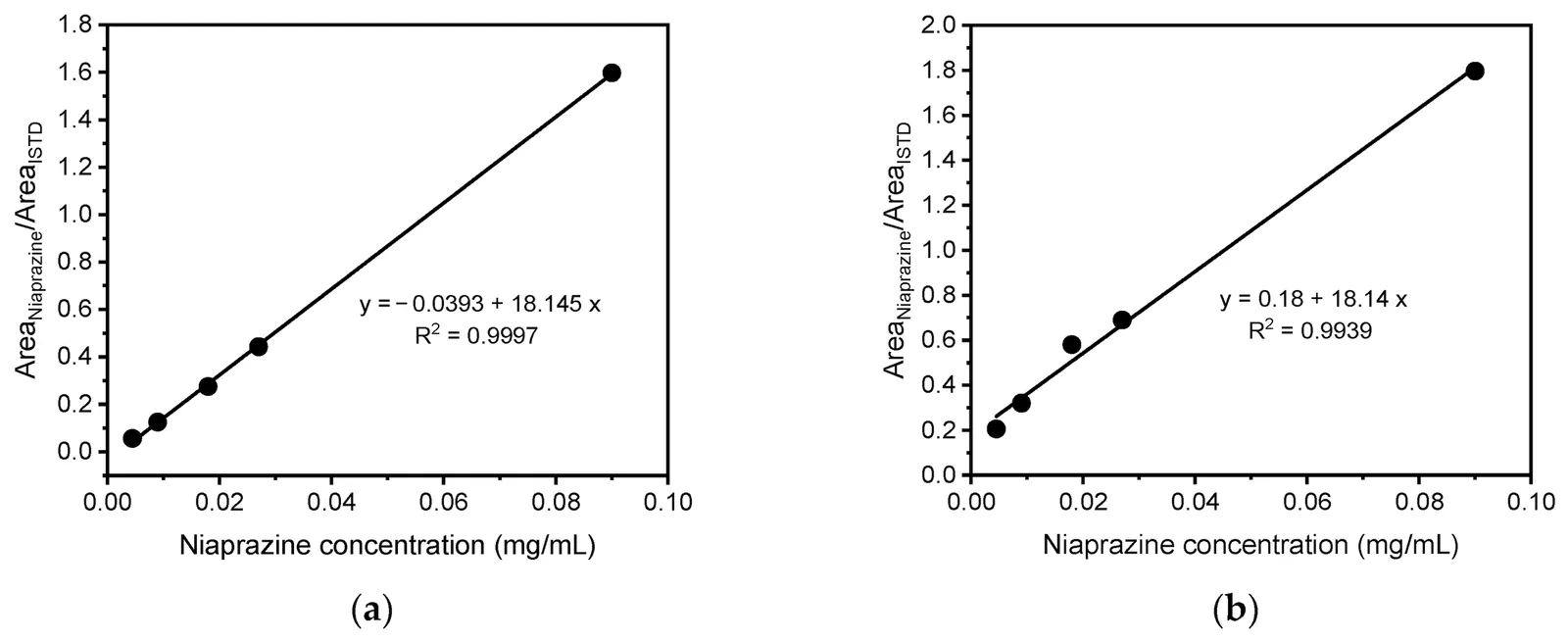
Calibration curve of niaprazine obtained by UV detection at 254 nm (**a**) and MS detection (TIC) (**b**). The response ratio (A_Niaprazine_/A_ISTD_) for each calibration point represents the mean of three independent measurements.

Each calibration point represents the mean value of three independent measurements. Calibration curves obtained using UV detection (254 nm) and MS detection (Total Ion Current, TIC) are reported in Figure 3a,b, respectively. The observed standard deviation ranged from 0.002 to 0.04 for UV detection and from 0.01 to 0.04 for MS detection, indicating good repeatability of the method.

### 2.7. Physical-Chemical Stability Study

A comprehensive physicochemical stability study was conducted on the formulations. Each sample, prepared at a concentration of 3 mg·mL^−1^ of niaprazine was stored in tightly sealed pharmaceutical-grade 30 mL amber glass bottles equipped with certified screw-cap closures suitable for non-sterile oral liquid preparations, protected from light, under three different storage conditions: 4–8 °C in a refrigerator, 25 °C in a thermostatic chamber, and 40 °C/75% RH in a climatic chamber (Climacell; MMM Medcenter, Munich, Germany). The accelerated storage condition (40 °C/75% RH) was selected to simulate reasonably foreseeable worst-case environmental conditions during storage. Because the bottles remained tightly sealed throughout the study, the controlled relative humidity affected only the external storage environment and did not directly influence the formulation inside the containers. During a 12-month period, the chemical stability of niaprazine was evaluated by the HPLC methods described above, quantifying the residual drug content and the possible presence of degradants at several time points (0, 1, 3, 6, 9, and 12 months). The amount of API was assessed by analyzing chromatogram peak areas in relation to the drug calibration curve. Aliquots of 0.5 mL of each sample were taken and diluted 1:100 with mobile phase in a volumetric flask. The diluted sample was vigorously mixed for 1 min and placed in an ultrasonic bath for 5 min. After this procedure, 1 mL of each sample was diluted 1:10 with mobile phase in a volumetric flask and then analyzed by HPLC. After each sampling, the bottles were immediately recapped and returned to their respective storage conditions. Chemical stability of niaprazine in the formulations stored at each temperature after 12 months was also evaluated by the method described in Section 2.6. *High-performance liquid chromatography coupled with diode array detection and mass spectrometry (HPLC–DAD–MS)*. Briefly, a volume of 0.1 mL of niaprazine syrup formulation stored at each temperature and 0.3 mL of trazodone hydrochloride stock solution (1.255 mg·mL^−1^) were transferred to a 10 mL volumetric flask and diluted to volume with the mobile phase. The diluted samples were vigorously mixed for 1 min and placed in a ultrasonic bath for 5 min. After this procedure, 1 mL of each sample was diluted 1 to 10 with mobile phase in a volumetric flask and then analyzed by HPLC. To confirm the physical stability of the formulation stored under the same conditions, its appearance (color, clarity, presence of visible particulates, odor) and pH values were monitored at each time point defined.

### 2.8. Microbiological Analysis

The microbiological quality of the niaprazine syrup formulation stored at 4–8 and 25 °C was also evaluated in accordance with the European Pharmacopoeia (12th Edition) requirements for non-sterile oral liquid preparations [21]. Microbial contamination was assessed using the plate count method for the determination of the total aerobic microbial count (TAMC) and total yeast and mold count (TYMC). Petri dishes (9 cm diameter) were prepared by pouring approximately 20 mL of molten agar medium at 45 °C. Mueller–Hinton agar was used for bacterial growth and Sabouraud dextrose agar for fungal growth. After solidification of the agar, 0.1 mL of the sample, previously diluted 1:10 (*v*/*v*) in sodium chloride-peptone buffer (pH 7.0), was evenly spread onto the agar surface. The plates were incubated at 37 °C, and colony counting was performed after 1 and 5 days. Analyses were carried out in triplicate at predefined time points (t = 0, 1 and 2 months) on three independent batches of the formulation. Method suitability testing was performed by inoculating the diluted formulation with known low levels of reference microorganisms (*Escherichia coli* ATCC 25922 and *Candida albicans* ATCC 10231) and comparing microbial recovery with control media, confirming the absence of inhibitory effects of the formulation. In parallel, microbial load was also evaluated using the Most Probable Number (MPN) method as a complementary technique [22]. Three serial dilutions of each sample were prepared in sodium chloride-peptone buffer, and three aliquots of 1 mL from each dilution were inoculated into test tubes containing 9 mL of liquid culture medium (Mueller–Hinton broth for bacteria and Sabouraud dextrose broth for fungi). The inoculated tubes were incubated at 37 °C for 5 days and visually inspected for evidence of microbial growth (turbidity). Negative controls (culture medium only) and positive controls inoculated with *Escherichia coli* ATCC 25922 (bacteria) and *Candida albicans* ATCC 10231 (fungi) were included to confirm the validity of the method. Microbiological acceptability was assessed according to the European Pharmacopoeia limits for oral preparations (EP 5.1.4), including TAMC ≤ 10^3^, TYMC ≤ 10^2^, and the absence of specified microorganisms (*Escherichia coli*: absence/1 g o 1 mL). All samples complied with the acceptance criteria throughout the study period under the tested storage conditions.

### 2.9. Statistical Analysis

Quantitative data are expressed as mean ± standard deviation (SD) of three independent determinations. Statistical analysis was performed on the residual niaprazine concentrations determined for the compounded syrup formulation after 12 months of storage under the three investigated temperature conditions (4–8, 25, and 40 °C). Differences among storage conditions were evaluated using one-way analysis of variance (ANOVA), followed by Tukey’s Honest Significant Difference (HSD) post hoc test for multiple comparisons. Tukey’s test was selected to identify pairwise differences among storage conditions when the overall ANOVA indicated statistical significance. Statistical analyses were performed using OriginPro 2021 (OriginLab Corporation, Northampton, MA, USA). Differences were considered statistically significant at *p* < 0.05.

## 3. Results and Discussion

Currently, no commercially available liquid formulation of niaprazine exists for the effective management of sleep disorders, particularly in pediatric and geriatric patients, making the preparation of magistral formulations in pharmacy practice necessary. In accordance with good compounding practice, the recommended BUD for such extemporaneous preparations is generally limited to 30 days. This constraint represents a practical challenge, as it requires frequent renewal of the formulation and may negatively impact therapeutic continuity, healthcare costs, and patient adherence. To address these limitations, the present study evaluated the chemical, physical and microbiological stability of a magistral oral formulation of niaprazine. In parallel, the study explored the use of selected ready-to-use compounding vehicles as a complementary approach, with the aim of assessing their suitability as potential alternatives in routine pharmacy practice. Chemical stability was assessed using HPLC-DAD and -MS analytical techniques, while visual inspection and pH measurements were performed in parallel to monitor the physical integrity of the formulation over time. The stability data indicate that the syrup formulation maintains acceptable physicochemical properties beyond the currently recommended timeframe, providing a scientifically sound basis for considering an extension of BUD. In addition, microbiological stability studies were conducted for the syrup formulation stored at 4–8 and 25 °C, conditions under which chemical and physical stability were most favorable. Over a two-month observation period, the results confirmed compliance with pharmacopeial acceptance criteria, further supporting the overall stability of the niaprazine syrup. Similarly, the ready-to-use vehicles provided comparable chemical and physical stability under the investigated conditions, suggesting that they may represent standardized and practical alternatives for routine compounding, while complementing rather than replacing individualized magistral formulations.

### 3.1. Preparation of Niaprazine Syrup Formulation

The qualitative and quantitative composition of the niaprazine oral solution was designed to obtain a target concentration of 3 mg·mL^−1^, while ensuring adequate chemical, physical, and microbiological stability, together with acceptable palatability and dose flexibility. Oral niaprazine is typically administered at low, individualized doses, commonly ranging from 0.5 to 1 mg·kg^−1^ in pediatric patients and 5–20 mg per day in geriatric subjects, thereby often requiring flexible dosing strategies achievable through compounded liquid formulations [23]. The concentration of 3 mg·mL^−1^ was selected as a practical compromise that allows accurate dose individualization in clinical practice, particularly for pediatric and geriatric patients, without exceeding the solubility limits of the API in an aqueous environment. Niaprazine exhibits low intrinsic solubility in water, reported in the literature to be below 1 mg·mL^−1^ at neutral pH [24], which would be insufficient to achieve the desired concentration without formulation optimization. The inclusion of tartaric acid was therefore essential to adjust the formulation to a mildly acidic pH (approximately 5.4). In the presence of tartaric acid, niaprazine undergoes protonation at the tertiary piperazine nitrogen and may form a water-soluble tartrate salt, thereby markedly enhancing its aqueous solubility and contributing to pH control and formulation stability. Potassium sorbate was selected as a preservative due to its well-established antimicrobial activity, particularly against yeasts and molds, and its optimal performance in acidic environments [25]. At the selected pH value, this excipient contributes effectively to microbiological stability without significantly affecting taste or chemical compatibility. Its use is widely accepted in oral liquid formulations and aligns with pharmacopeial recommendations for non-sterile aqueous preparations. The choice of simple syrup as the main vehicle was driven by both technological and patient-related considerations. From a formulation standpoint, the high sucrose content reduces water activity, thereby complementing the preservative system and contributing to microbiological stability [26]. In addition, syrup provides suitable viscosity, improving dose uniformity and ease of administration. From a patient perspective, syrup is a well-established taste-masking vehicle, capable of mitigating the potentially unpleasant taste associated with piperazine-containing psychoactive compounds, where palatability is a critical determinant of treatment adherence [27]. Purified water was used to adjust the final volume, ensuring compliance with pharmacopeial quality requirements and reproducibility of the preparation.

### 3.2. Forced Degradation Studies

**Figure 4:**
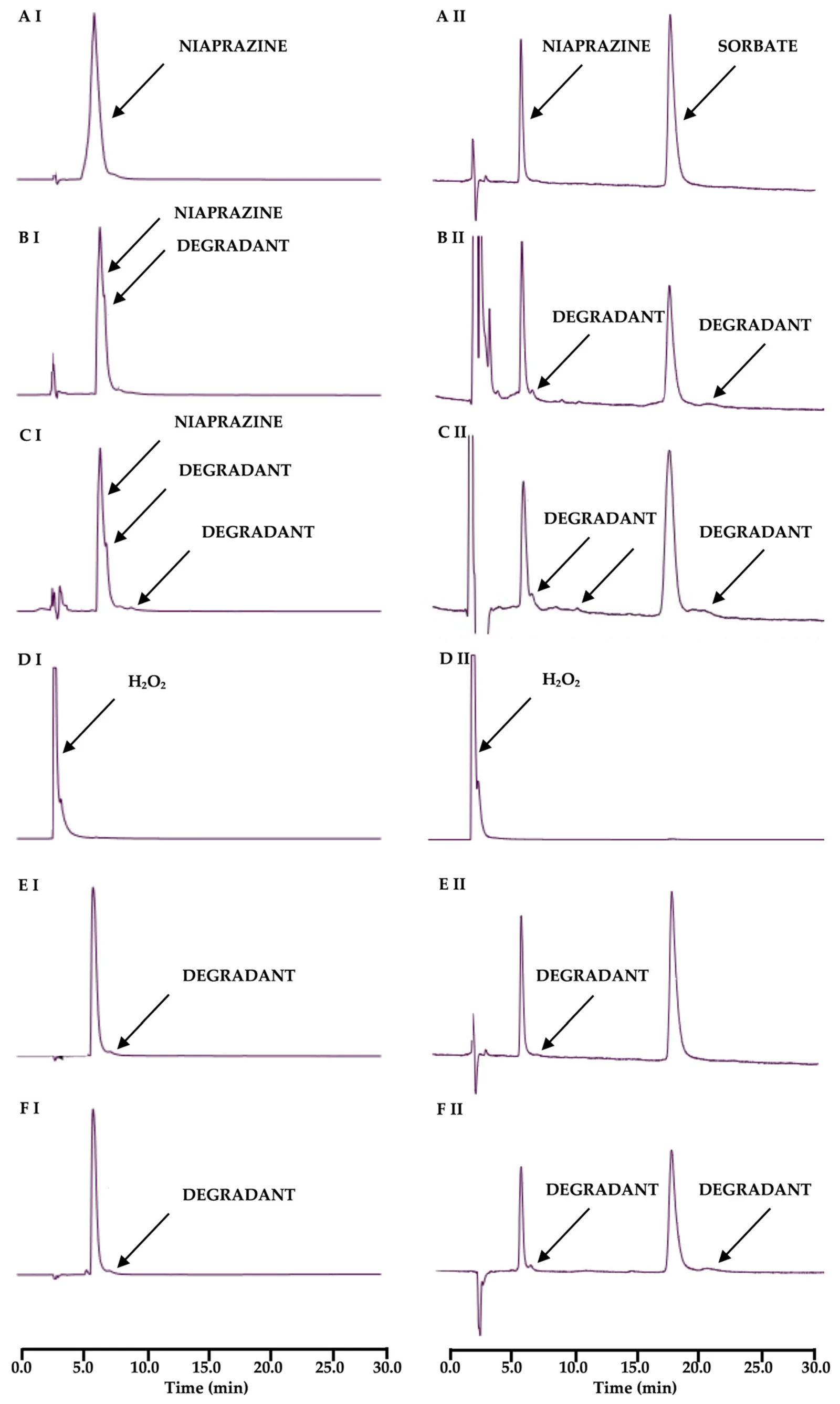
Representative HPLC chromatograms (λ = 245 nm) of niaprazine in methanol (1 mg·mL^−1^) (**A**(I)) and syrup formulation (3 mg·mL^−1^) (**A**(II)). Chromatograms of the API subjected to stress test in presence of HCl (**B**(I,II)), NaOH (**C**(I,II)), H_2_O_2_ (**D**(I,II)), high temperature (70 °C) (**E**(I,II)) and UV-light (λ = 365 nm) (**F**(I,II)) for 72 h (for 1 h under oxidative stress condition).

Forced degradation studies were conducted on both the API and the syrup formulation under acidic, alkaline, oxidative, thermal, and photolytic stress conditions to evaluate the ability of the developed HPLC-DAD method to distinguish the parent drug from excipients and potential degradation products, thereby confirming its suitability as a stability-indicating method. Representative chromatograms of niaprazine in methanol and the syrup formulation, together with the corresponding chromatograms under each stress condition, are shown in Figure 4A–F(I,II).

The representative chromatogram of the niaprazine syrup formulation (Figure 4A(II)) shows the presence of two well-resolved major peaks, with the first eluting at approximately 6.8 min, corresponding to the API, and a second peak eluting at around 18.6 min, attributable to the potassium sorbate. The clear chromatographic separation between the drug and the preservative confirms the suitability of the method for monitoring both components within the formulation.

Building on this chromatographic profile, forced degradation studies demonstrated that, under all applied stress conditions, the developed HPLC-DAD method was capable of distinguishing the characteristic niaprazine peak from newly formed chromatographic peaks attributable to degradation products potentially originating from both the active substance and the formulation excipients. In particular, acidic and alkaline conditions led to the formation of multiple additional peaks. These reflected the presence of niaprazine-related degradation products as well as potential excipient-derived species, while the parent compound remained clearly identifiable (Figure 4B,C(I,II)). In contrast, oxidative stress resulted in the complete degradation of niaprazine, with the parent peak no longer detectable in the presence of peroxide (H_2_O_2_) (Figure 4D(I,II)). Conversely, thermal and photolytic stress produced only limited changes in the chromatographic profiles, with the niaprazine peak largely preserved and only minor secondary signals observed (Figure 4E,F(I,II)). The use of a diode array detection proved particularly advantageous for the analysis of the niaprazine syrup formulation, as it enabled confirmation of the identity of the API peak through full UV spectral matching and assessment of peak purity. The results (Table 5) supported the selectivity of the method and its suitability as a stability-indicating approach for the chemical stability evaluation of the niaprazine syrup formulation.

To further assess the suitability of the HPLC-DAD method as a stability-indicating method across different excipient matrices, the chromatographic behavior of niaprazine was also evaluated in the three commercially available ready-to-use oral vehicles characterized by similar, although not identical, excipient compositions. Under all tested stress conditions, no co-eluting peaks were observed at the retention time of niaprazine, and the degradation products generated from either the API or the vehicle excipients remained chromatographically resolved from the parent compound (Table 5). These findings indicate that the method is sufficiently selective to distinguish niaprazine from matrix-related components and their potential degradation products, supporting its applicability to the different oral liquid vehicles evaluated in this study.

### 3.3. Physicochemical Stability Study of Niaprazine Syrup Formulation

The stability of the extemporaneous niaprazine syrup was investigated through a stepwise analytical approach combining HPLC-DAD and LC-MS, with the objective of assessing the chemical robustness of the formulation over time.

An initial chemical stability study was conducted for up to 9 months using HPLC-DAD, focusing on monitoring the niaprazine chromatographic peak area over time. This approach allowed the evaluation of potential changes in drug content as well as the detection of any additional chromatographic peaks that could be attributed to degradation products. The selection of the three temperatures 4–8, 25, and 40 °C as storage conditions for the physicochemical stability study of the oral liquid formulation was based on regulatory guidance and pharmaceutical practice and was intended to provide a comprehensive evaluation of the behavior of the formulation under real-world and stressed conditions [27]. Storage at 4–8 °C represents refrigerated conditions, which are commonly recommended for aqueous oral formulations to limit chemical degradation and microbial proliferation, particularly for extemporaneous preparations. The temperature of 25 °C reflects standard room temperature conditions, corresponding to typical storage in a pharmacy and patient use, and is therefore essential for assessing stability under routine handling and administration conditions. Finally, 40 °C was selected as an accelerated testing condition, in line with international stability-assay guidelines (e.g., ICH Q1A), to evaluate the robustness of the formulation and to identify potential degradation pathways that may not be evident under normal storage. In addition to its role as an accelerated stress condition, 40 °C represents an extreme but realistic temperature that pharmaceutical products may encounter during storage or transport, including non-climate-controlled environments, temporary exposure during distribution, or storage in low-resource locations.

As reported in Table 6, niaprazine concentrations remained close to the nominal value of 3.0 mg·mL^−1^ throughout the 9-month study at all storage temperatures, with all results falling within the commonly accepted pharmacopeial acceptance range of 90–110% (i.e., 2.70–3.30 mg·mL^−1^). At 4–8 and 25 °C, only minor fluctuations were observed over time, indicating a favorable stability profile under refrigerated and room-temperature storage. At 40 °C, a modest reduction in niaprazine concentration was detected at later time points (down to 2.88 ± 0.14 mg·mL^−1^ at 9 months, ~96% of nominal), suggesting the onset of accelerated instability under stressed storage. Importantly, the identity and peak purity of the niaprazine signal were assessed using DAD by comparing the UV-Vis spectrum across the peak, confirming that no co-eluting species interfered with niaprazine quantification. In parallel, chromatographic evaluation at 40 °C revealed the appearance of minor additional peaks, attributable to the formation of degradation products, supporting the stability-indicating capability of the analytical method. Overall, these results indicate that niaprazine retains acceptable chemical stability in the syrup matrix across the tested conditions, with the most robust profile observed at 4–8 and 25 °C, while 40 °C may promote limited degradation over prolonged storage.

**Figure 5:**
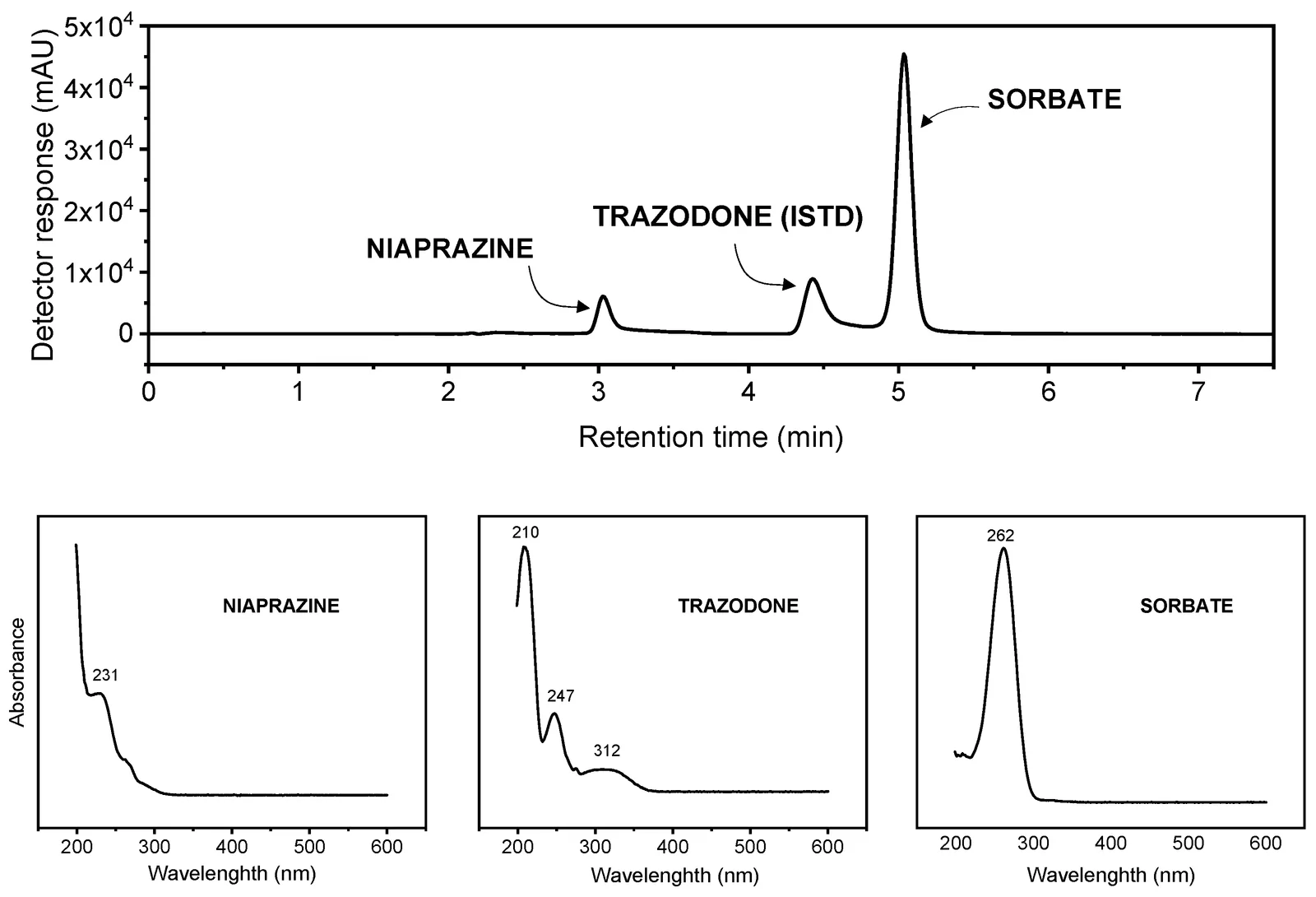
Representative HPLC chromatographic profile (λ = 254 nm) of the niaprazine syrup formulation stored for 12 months at 25 °C. The signals eluting at 3.02, 4.42, and 5.02 min are attributed to niaprazine, trazodone (internal standard, ISTD), and sorbate, respectively. Corresponding UV–Vis spectra (200–600 nm) of the three peaks at 3.02, 4.42, and 5.02 min, assigned to niaprazine, trazodone (ISTD), and sorbate, respectively.

To further support the chemical stability assessment, an additional study was performed on the same formulation at 12 months using the quantitative HPLC–DAD–MS method described in Section 2.6 with internal standardization. In this phase, trazodone hydrochloride (Figure 2) was employed as an internal standard to improve analytical accuracy and compensate for potential matrix effects. The combined use of DAD-MS detection provided a comprehensive and reliable analytical platform for the qualitative and quantitative characterization of niaprazine in the syrup formulation. As shown in Figure 5, the HPLC–DAD chromatographic profile recorded at 254 nm enabled clear separation of the main formulation components, with well-resolved peaks corresponding to niaprazine, trazodone (internal standard, ISTD), and sorbate. The acquisition of full UV–Vis spectra (200–600 nm) by diode array detection further confirmed peak identity and excluded co-elution phenomena, supporting the selectivity of the method.

**Figure 6:**
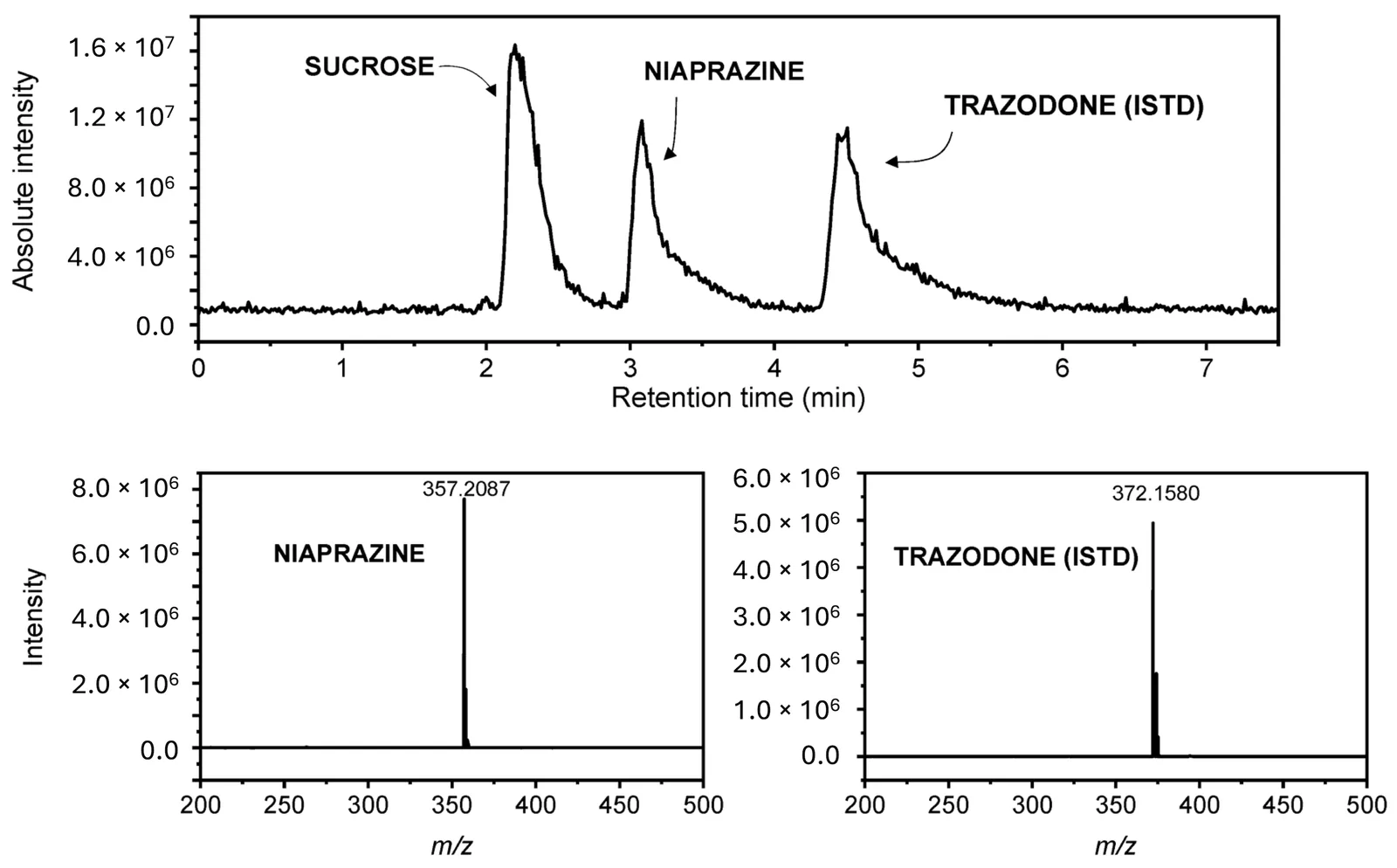
Representative HPLC–MS total ion chromatogram (TIC) of the niaprazine syrup formulation stored for 12 months at 25 °C. The signals eluting at 2.22, 3.09, and 4.48 min are attributed to sucrose, niaprazine, and trazodone (internal standard, ISTD), respectively. The corresponding mass spectra of the peaks at 3.09 and 4.48 min, assigned to niaprazine ([M + H]^+^ *m*/*z* 357.2087) and trazodone ([M + H]^+^ *m*/*z* 372.1580), respectively.

Complementary HPLC-MS analysis in TIC mode enabled unequivocal assignment of the chromatographic signals, including the detection of sucrose at early retention times and confirmation of niaprazine and trazodone by their characteristic protonated molecular ions [M + H]^+^ at *m*/*z* 357.2087 and 372.1580, respectively (Figure 6). The combination of chromatographic resolution and mass spectral information ensured clear discrimination between the active compound, the internal standard, and the excipients, even within a complex syrup matrix.

Trazodone was selected as an internal standard due to its structural features and similar ionization behavior as niaprazine under positive electrospray ionization (ESI^+^), while remaining both chromatographically and spectrometrically distinguishable. This enabled reliable correction for matrix effects and analytical variability, thereby ensuring robust and reproducible quantification of niaprazine in the formulation under different storage conditions.

Table 7 summarizes the data from HPLC-DAD-MS analysis of syrup samples stored at 4–8, 25, and 40 °C for 12 months. The decrease in niaprazine concentration upon storage at 40 °C appears statistically significant compared to room temperature (25 °C) conditions, as revealed by Tukey’s test on both sets of data obtained by DAD (*p* = 0.011) and MS (*p* = 0.035) detection. Although minor additional chromatographic peaks were observed by HPLC-DAD, no corresponding ionizable species could be reliably detected or identified as degradation products under the LC–MS conditions, despite the enhanced selectivity provided by the mass spectrometer. Importantly, all assay values remained within the stability acceptance limits specified by the Pharmacopoeia (±10%), supporting the overall chemical stability of niaprazine in the syrup matrix over long-term storage.

Visual inspection was performed alongside the chemical stability assessment to evaluate the physical stability of the syrup formulations stored at 4–8, 25, and 40 °C over 12 months. Throughout the study period, all samples remained clear and homogeneous, with no evidence of turbidity, phase separation, or precipitation. pH values remained stable across all storage conditions, showing only minor fluctuations (≤0.5 pH units), which were considered acceptable and not indicative of physicochemical instability. At 40 °C, a visually observable color change was noted at the end of the storage period, while the formulation remained clear and free of visible particulates. This color change may plausibly be related to temperature-driven sucrose inversion, generating glucose and fructose that can subsequently promote non-enzymatic browning phenomena within the syrup vehicle. Overall, these findings support the conclusion that the formulation retained its physical integrity during long-term storage, including under accelerated temperature conditions.

### 3.4. Preparation of Niaprazine Formulations in Ready-to-Use Bases and Stability Studies

**Figure 7:**
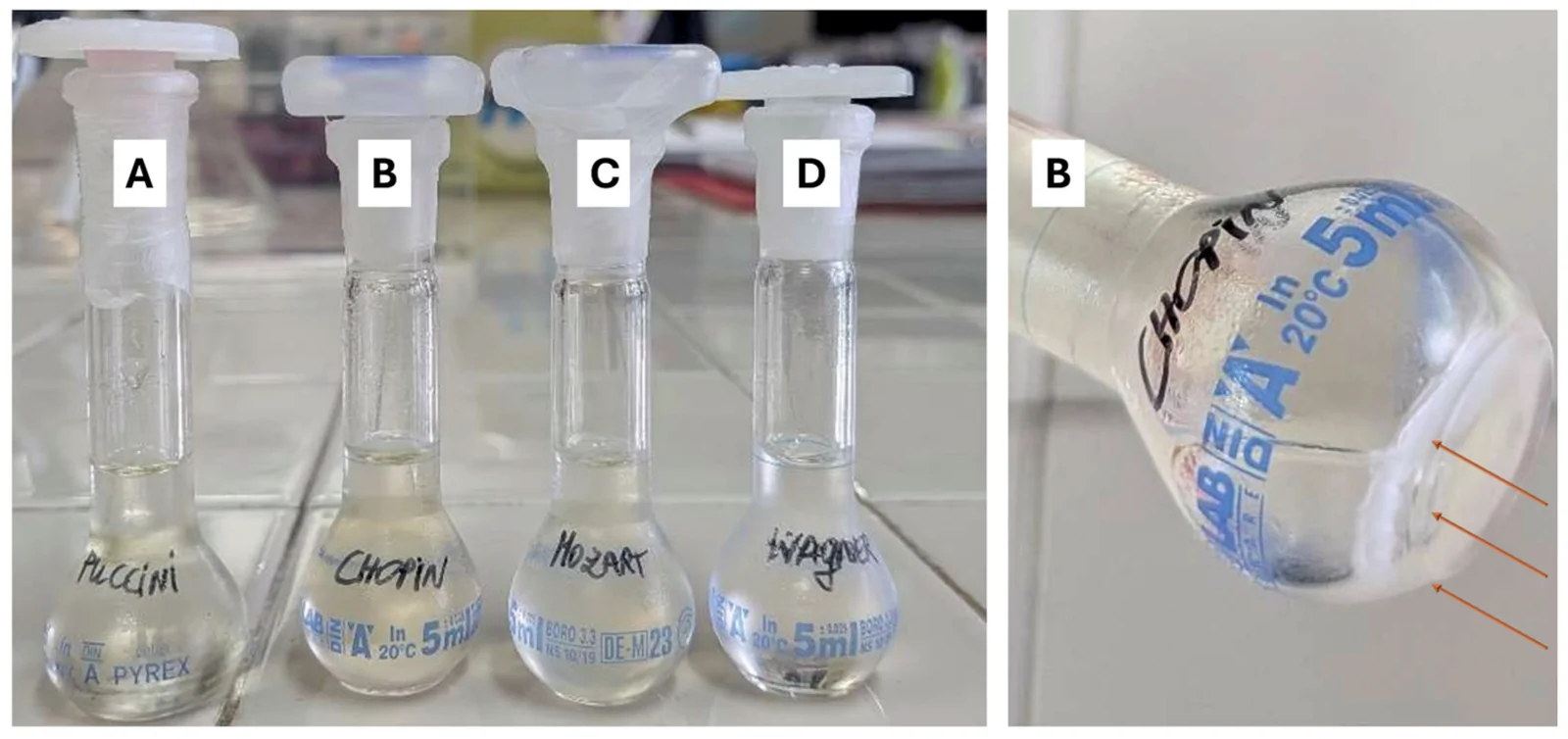
Visual appearance of niaprazine 3 mg·mL^−1^ formulations prepared in the four ready-to-use liquid vehicles Puccini (**A**), Chopin (**B**), Mozart sugar free (**C**) and Wagner (**D**) after one week of storage at 25 °C (left panel). The right panel highlights the presence of visible precipitation in formulation prepared using the Chopin vehicle.

The different capacities of the four ready-to-use vehicles to maintain niaprazine in a visually homogeneous solution can be mainly explained by the pH of the formulations and the possibility of salt formation. Niaprazine is a weak base (pKa ≈ 7.9), and its solubility increases upon protonation. In acidic environments such as Puccini (pH 4.0–4.5) and Mozart sugar-free (pH ≈ 4.5), the presence of citric acid (pKa_1_ ≈ 3.1; pKa_2_ ≈ 4.8) ensures a favorable difference between the pKa of the acid and that of the base (ΔpKa ≥ 2), thereby promoting the protonation of the drug and the formation of water-soluble salts. This explains the formation of clear and homogeneous formulations at the target drug concentration of 3 mg·mL^−1^. A similar behavior is observed in the Wagner vehicle (pH 5.5–5.9), where the citrate buffer system still provides conditions that promote partial protonation of niaprazine, allowing the preparation of a clear and homogeneous formulation (Figure 7, samples A, C and D on the left). In this case, hydroxypropyl-β-cyclodextrin may also contribute to drug solubilization through inclusion complex formation [28], although this effect is likely secondary to pH-dependent ionization.

In contrast, in the Chopin vehicle (pH 8.0–9.0), the alkaline conditions do not favor protonation, and the ΔpKa with the available buffering species (sodium carbonate) is not sufficient to promote salt formation. As a result, niaprazine remains predominantly in its non-ionized form, which is poorly soluble in aqueous media. Although hydroxypropyl-β-cyclodextrin is present, its effect is not sufficient to compensate for the lack of salt formation, resulting in the presence of visible undissolved drug and subsequent precipitation (Figure 7, sample B on the right).

Based on these preliminary studies, chemical stability of the drug and physical stability of the formulations prepared with the three ready-to-use vehicles (Puccini, Mozart sugar-free, and Wagner) were evaluated. The results indicate that the formulations exhibited a good chemical stability profile, with API concentrations remaining close to the nominal value (3 mg·mL^−1^) throughout the study period under all tested storage conditions. The observed variations over time were minimal and within the expected analytical variability, with no evidence of significant degradation of the active compound under the investigated conditions (Table 8).

In particular, high stability was maintained up to 12 months in both the Puccini and Mozart sugar-free formulations, whereas the Wagner formulation showed a similar stability profile over a shorter observation period (3 months at 4–8 °C and 2 months at 25 and 40 °C). Even under accelerated conditions at 40 °C, niaprazine showed only a modest decrease in concentration over time, suggesting limited temperature-dependent degradation (Table 8 and Table 9).

Visual inspection was performed in parallel with the chemical stability assessment to evaluate the behavior of the formulations stored at 4–8, 25, and 40 °C over observation periods of 2 and 12 months. Throughout the study, all niaprazine formulations prepared with Puccini, Mozart sugar-free, and Wagner vehicles remained clear and homogeneous, with no evidence of turbidity, phase separation, or precipitation. Their pH values remained stable across all storage conditions, showing only minor fluctuations below 0.5 pH units. The limited pH variation throughout the study indicates that the acidic environment was maintained during storage. This finding is particularly relevant because maintenance of a mildly acidic environment contributes both to preserving niaprazine in its protonated, soluble form and to supporting the antimicrobial effectiveness of potassium sorbate.

### 3.5. Microbiological Analysis

Microbiological quality was evaluated in a separate experimental study specifically designed to assess the microbiological stability of the compounded niaprazine syrup during a two-month observation period under refrigerated and room-temperature storage conditions. This investigation complemented the long-term physicochemical stability study, which was independently conducted over 12 months using validated chromatographic methods. The microbiological quality of the niaprazine syrup formulations was evaluated using pharmacopeial methods on three independent formulations (samples 1–3) prepared in three independent production batches and stored under controlled conditions. Plate count analyses performed in accordance with the European Pharmacopoeia demonstrated the absence of detectable bacterial and fungal growth in all samples throughout the study period. These findings were consistent across all tested time points and storage conditions (4–8 and 25 °C), confirming compliance with the microbiological acceptance criteria for non-sterile oral liquid preparations.

Microbial load was further assessed using the Most Probable Number (MPN) method, which corroborated the plate count results. For all formulations, total aerobic microbial counts remained ≤ 10^3^ CFU·mL^−1^ and total yeast and mold counts ≤ 10^2^ CFU·mL^−1^ over the entire observation period. Moreover, no specified microorganisms, including *Escherichia coli*, were detected in any of the analyzed samples. Taken together, these results demonstrate that the niaprazine syrup complied with the predefined microbiological acceptance criteria for up to two months under the tested storage conditions (Table 10).

In addition to microbiological evaluation, pH measurements were performed at predefined time points (Table 10) to monitor potential physicochemical changes associated with degradation phenomena or microbial growth. Across all formulations, pH values remained essentially unchanged compared with baseline (approximately 5.4), irrespective of storage temperature or duration. Variations were consistently limited within 0.5 pH units during the two-month observation period at 4–8 and 25 °C. The maintenance of a stable pH profile is a relevant indicator of chemical stability and indirectly supports the microbiological findings. Visual inspection was conducted in parallel to assess physical stability. Throughout the study period, all samples remained clear and homogeneous, with no evidence of color change, turbidity, precipitation, phase separation, or odor development. These observations indicate that the formulations preserved their macroscopic integrity under the tested storage conditions.

Taken together with the microbiological results, the stable pH profile and the absence of visible alterations support the conclusion that the niaprazine syrup maintained both physicochemical and microbiological stability over the two-month storage period at 4–8 and 25 °C. These findings are consistent with the longer-term stability trend and suggest that the current recommended BUD may warrant reconsideration under standard storage conditions.

The present study was designed to test the working hypothesis that a rationally formulated, mildly acidic sucrose-based niaprazine syrup could provide adequate chemical, physical, and microbiological stability beyond the conservative 30-day BUD typically assigned to compounded aqueous oral preparations. In this context, the study was also designed to explore the feasibility of using ready-to-use compounding vehicles as a complementary approach to conventional extemporaneous formulations. This work extends the currently available evidence on compounded niaprazine oral liquid formulations. To date, published studies have primarily focused either on short-term physicochemical stability of magistral niaprazine syrups or on the development of alternative oral dosage forms to improve patient acceptability. Consequently, a comprehensive evaluation integrating long-term physicochemical stability, microbiological quality, stability-indicating forced degradation studies, and the assessment of commercially available ready-to-use compounding vehicles has not previously been reported [29,30].

This first hypothesis was supported by the overall stability profile, as niaprazine assay values remained within pharmacopeial acceptance limits under routine storage conditions, with no clinically meaningful physicochemical changes observed over time. These findings are consistent with previous studies on compounded niaprazine oral liquids, in which maintenance an acidic environment through the addition of an organic acid was considered essential to ensure complete drug solubilization and acceptable physicochemical stability during storage. However, unlike previous investigations, the present study substantially extends the available evidence by demonstrating long-term physicochemical stability over 12 months, together with microbiological evaluation, stability-indicating forced degradation studies, and orthogonal analytical confirmation by LC-MS [29]. From a broader perspective, these results are consistent with the general evidence that appropriate control of pH and preservative systems can markedly improve the stability of compounded oral formulations, particularly when prepared using suitable vehicles. In the present formulation, maintaining a mildly acidic pH ensured that niaprazine remained predominantly in its protonated form, thereby enhancing its aqueous solubility and minimizing the risk of precipitation throughout storage. This formulation strategy is consistent with previous reports on compounded niaprazine, in which acidification was intentionally introduced to improve drug solubility and formulation stability [29]. The temperature-dependent trend observed under accelerated storage is consistent with established principles of stress testing, supporting the use of 40 °C as a conservative condition to simulate worst-case scenarios rather than routine conditions. Importantly, the concordance between HPLC–DAD and LC–MS analyses of the sucrose-based niaprazine syrup formulation confirms the reliability of drug quantification within a complex matrix and suggests that any degradation occurring at elevated temperature is limited and/or remains below the threshold for unequivocal identification under the applied MS conditions. Furthermore, the orthogonal confirmation provided by LC-MS reduces the likelihood of co-eluting degradation products interfering with UV-based quantification, thereby strengthening confidence in the long-term stability assessment of the formulation. A central implication of this work is its relevance for patient management and pharmacy. Extending the period of use for a stable niaprazine syrup has the potential to reduce the burden of frequent refilling, support continuity of therapy, and improve adherence, particularly in pediatric and geriatric patients where dose individualization is essential. The importance of providing robust and standardized pharmaceutical formulations is further reinforced by the renewed pharmacological interest in niaprazine reported in recent years, highlighting the continuing clinical relevance of this active substance and the need for evidence-based compounded formulations [18]. Based on the combined chemical, physical, and microbiological evidence, the data provide a scientifically sound rationale to support a potential extension of the BUD to up to two months when stored at 4–8 °C or 25 °C, provided that preparation and handling follow good compounding practice. At the same time, a cautious interpretation remains appropriate, as microbiological testing beyond two months and under simulated in-use conditions was not investigated in the present study and may represent a critical determinant for further extension. Regarding the second objective, this study demonstrated that commercially available ready-to-use compounding vehicles can represent a viable alternative to traditional magistral syrup formulations for the preparation of niaprazine oral liquids. Three of the four tested vehicles maintained the aqueous solubility, chemical stability, and physical stability of niaprazine under the investigated storage conditions, showing performance comparable to that of the syrup formulation. Extended stability was demonstrated for selected vehicles, with Puccini and Mozart sugar-free bases maintaining acceptable drug content and formulation stability for up to 12 months at 4–8, 25 and 40 °C. Likewise, Wagner vehicle maintained acceptable chemical and physical stability throughout the entire investigated period (2 months) under all the tested storage conditions. These findings are particularly relevant because the investigated vehicles differ in excipient composition, buffering systems, sweeteners, viscosity modifiers, and preservative blends, yet they provided comparable stability profiles. This observation suggests that niaprazine stability is not restricted to a single formulation design but can be successfully maintained in different appropriately designed oral liquid matrices. Recent research has also explored alternative niaprazine formulations aimed at improving patient acceptability, such as xanthan gum-based oral gels [30]. While these approaches pursue different formulation objectives, the present results demonstrate that ready-to-use oral liquid vehicles represent a complementary strategy that preserves the advantages of conventional liquid dosage forms while improving formulation standardization and reproducibility during routine compounding. Although these findings do not replace the rationale for a well-designed magistral formulation, they suggest that commercial vehicles, characterized by optimized buffering systems, viscosity modifiers, and preservative blends, may represent a practical and standardized alternative for routine compounding practice. In addition, these vehicles may reduce preparation variability and simplify the compounding process, thereby improving reproducibility in pharmacy practice. Furthermore, the availability of a sugar-free option, such as the Mozart sugar-free vehicle, represents a relevant advantage, particularly for diabetic patients or those requiring controlled carbohydrate intake. Overall, the present investigation provides the most comprehensive stability evaluation currently available for compounded niaprazine oral liquid formulations, integrating long-term physicochemical stability, microbiological quality, stability-indicating forced degradation studies, orthogonal LC-MS confirmation, and the evaluation of commercially available ready-to-use compounding vehicles. Future research should therefore focus on strengthening translational applicability through extended microbiological monitoring beyond two months, including in-use simulations with repeated opening and dosing, evaluation of container–closure effects, and light exposure during handling. Collectively, these investigations would provide a more comprehensive evidence base for establishing risk-based BUDs and further support the implementation of stable compounded niaprazine oral liquid formulations in routine clinical practice.

## 4. Conclusions

The present study demonstrated that the compounded niaprazine syrup maintained its chemical and physical stability for up to 12 months under the investigated storage conditions, while microbiological quality remained compliant with the European Pharmacopoeia acceptance criteria throughout the two-month observation period. Taken together, these findings support extending the BUD of the compounded syrup to two months under the tested storage conditions, representing the longest period for which chemical, physical, and microbiological stability were all experimentally demonstrated. Such an extension may reduce the need for frequent reformulation, support therapeutic continuity, and improve patient adherence, particularly in pediatric and geriatric populations.

In parallel, the evaluation of selected ready-to-use compounding vehicles demonstrated that three of the tested vehicles were suitable for the preparation of homogeneous niaprazine oral liquid formulations and maintained chemical and physical stability during storage. Among them, the Puccini and Mozart sugar free vehicles exhibited physicochemical stability for up to 12 months under the investigated storage conditions. These ready-to-use vehicles may represent practical and standardized alternatives for routine compounding practice. However, as microbiological stability was not evaluated for these formulations, the present findings support only their long-term physicochemical stability, and no conclusions regarding an extended BUD can be drawn from this study.

## Figures and Tables

**Table 1:** Composition of niaprazine 3 mg·mL^−1^ syrup formulation.

Raw Material	Quantity
Niaprazine	0.45 g
Potassium Sorbate	0.30 g
Tartaric Acid	0.12 g
Simple Syrup	125 mL ^a^
Purified Water	q.s. ad 150 mL

^a^ Simple syrup prepared to contain 85% *w*/*v* sucrose.

**Table 2:** Composition and characteristics of Fast oral solution Puccini, Mozart sugar free, Wagner and Chopin ready-to-use bases.

Ready-to-Use Base	Qualitative Composition	pH
Puccini	Sucrose, glycerol, sorbitol, citric acid, disodium phosphate, potassium sorbate, highly purified water	4.0–4.5
Mozart sugar free	Glycerol, sorbitol, sucralose, xanthan gum, citric acid, sodium citrate, potassium sorbate, highly purified water	4.5
Wagner	Highly purified water, hydroxypropyl-β-cyclodextrin, sorbitol, vegetable glycerol, trisodium citrate dihydrate, sodium carboxymethylcellulose, citric acid, methylparaben, potassium sorbate, propylparaben, raspberry flavor	5.5–5.9
Chopin	Highly purified water, hydroxypropyl-β-cyclodextrin, sodium bicarbonate, sodium carboxymethylcellulose, methylparaben, propylparaben, sucralose, strawberry flavor	8.0–9.0

**Table 3:** Forced degradation conditions for niaprazine stock solution in methanol and its formulation.

Stress Type	Niaprazine Stock Solution or Formulation (mL)	Condition
Acidic	0.20	0.80 mL HCl 5 N, 72 h, 25 °C
Alkaline	0.20	0.80 mL NaOH 5 N, 72 h, 25 °C
Oxidative	0.20	0.80 mL H_2_O_2_ 30% *v*/*v*, 1 h, 25 °C
Photolytic	0.10	0.90 mL water, 72 h, 25 °C
Thermal	0.10	0.90 mL water, 72 h, 70 °C

**Table 4:** Composition of the niaprazine calibration standards.

Standard Solution	Stock Solution (mL)	Concentration (mg·mL^−1^)
1	0.05	0.0045
2	0.10	0.009
3	0.20	0.018
4	0.30	0.027
5	1.00	0.090

**Table 5:** Summary of forced degradation studies performed on niaprazine, the compounded syrup formulation, and the ready-to-use oral liquid vehicles (Puccini, Mozart Sugar Free, and Wagner). The table reports the chromatographic profile of the unstressed samples and the percentage of niaprazine remaining after exposure to acidic, alkaline, oxidative, photolytic and thermal stress conditions.

Condition	Experimental Condition	Niaprazine	Syrup Formulation	Puccini	Mozart Sugar Free	Wagner
		**Chromatographic Profile**				
Unstressed Control	No Stress	Single API Peak	Resolved Peaks	Resolved Peaks	Resolved Peaks	Resolved Peaks
		**Percentage of Remaining Niaprazine (%)**				
Acidic	5 N HCl, 72 h	52–56	73–82	79–85	75–88	83–89
Alkaline	5 N NaOH, 72 h	50–55	74–80	82–86	80–84	84–91
Oxidative	30% H_2_O_2_, 1 h	N.Q.	N.Q.	N.Q.	N.Q.	N.Q.
Photolytic	UV light (365 nm), 72 h	>98	>98	>98	>98	>98
Thermal	70 °C, 72 h	>98	>98	>98	>98	>98

N.Q., Not quantifiable.

**Table 6:** Residual niaprazine concentration (mg·mL^−1^) and drug content (% of theoretical concentration, calculated relative to the nominal niaprazine concentration of 3 mg·mL^−1^) in syrup formulations stored for 9 months at temperatures of 4–8, 25 and 40 °C. Concentrations and standard deviations (S.D.) were determined by HPLC analysis (λ = 245 nm) and represent the average of values obtained from three different experiments.

Temperature (°C)	Time (Month)	Niaprazine Concentration (mg·mL^−1^) and Drug Content (%) ± S.D.	
4–8	0	3.05 ± 0.20	101.67 ± 6.67
	1	2.97 ± 0.14	99.00 ± 4.67
	2	3.02 ± 0.13	100.67 ± 4.33
	3	3.04 ± 0.10	101.33 ± 3.33
	6	2.98 ± 0.22	99.33 ± 7.33
	9	3.13 ± 0.08	104.33 ± 2.67
25	0	3.05 ± 0.20	101.67 ± 6.67
	1	3.06 ± 0.18	102.00 ± 6.00
	2	3.08 ± 0.15	102.67 ± 5.00
	3	2.98 ± 0.21	99.33 ± 7.00
	6	2.95 ± 0.20	98.33 ± 6.67
	9	2.97 ± 0.11	99.00 ± 3.67
40	0	3.05 ± 0.20	101.67 ± 6.67
	1	3.08 ± 0.21	102.67 ± 7.00
	2	3.11 ± 0.19	103.67 ± 6.33
	3	2.94 ± 0.12	98.00 ± 4.00
	6	2.98 ± 0.13	99.33 ± 4.33
	9	2.88 ± 0.14	96.00 ± 4.67

**Table 7:** Residual niaprazine concentration (mg·mL^−1^) and drug content (% of theoretical concentration, calculated relative to the nominal niaprazine concentration of 3 mg·mL^−1^) in syrup formulation stored for 12 months at temperatures of 4–8, 25 and 40 °C. Concentrations and standard deviations (SD) were determined by HPLC analysis using diode array detection (DAD, λ = 254 nm) and mass spectrometric detection (MS, TIC mode). Data represent the mean of three independent experiments.

Temperature (°C)	Analytical Technique	Niaprazine Concentration (mg·mL^−1^) and Drug Content (%) ± S.D.	
4–8	HPLC-DAD	2.93 ± 0.01	97.67 ± 0.33
	HPLC-MS	2.99 ± 0.10	99.67 ± 3.33
25	HPLC-DAD	3.02 ± 0.02	100.67 ± 0.67
	HPLC-MS	3.10 ± 0.09	103.33 ± 3.00
40	HPLC-DAD	2.85 ± 0.08	95.00 ± 2.67
	HPLC-MS	2.85 ± 0.10	95.00 ± 3.33

**Table 8:** Residual concentration of niaprazine (mg·mL^−1^) and drug content (% of theoretical concentration, calculated relative to the nominal niaprazine concentration of 3 mg·mL^−1^) in the formulations prepared by using Puccini and Mozart sugar free vehicles stored for 12 months and Wagner vehicle stored for 3 months at temperatures of 4–8 °C and 2 months at 25 and 40 °C. Concentrations and standard deviations (S.D.) were determined by HPLC analysis (λ = 245 nm) and represent the average of values obtained from three different experiments.

Temperature (°C)	Time (Month)	Niaprazine Concentration (mg·mL^−1^) and Drug Content (%) ± S.D.					
		Puccini		Mozart Sugar Free		Wagner	
4–8	0	3.03 ± 0.10	101.00 ± 3.33	3.11 ± 0.16	103.67 ± 5.33	3.09 ± 0.21	103.00 ± 7.00
	1	2.97 ± 0.14	99.00 ± 4.67	3.17 ± 0.04	105.67 ± 1.33	2.97 ± 0.12	99.00 ± 4.00
	2	3.12 ± 0.23	104.00 ± 7.67	3.02 ± 0.13	100.67 ± 4.33	3.09 ± 0.23	103.00 ± 7.67
	3	2.94 ± 0.13	98.00 ± 4.33	3.09 ± 0.20	103.00 ± 6.67	3.01 ± 0.12	100.33 ± 4.00
	12	2.98 ± 0.21	99.33 ± 7.00	2.97 ± 0.14	99.00 ± 4.67	N.A.	N.A.
25	0	3.03 ± 0.10	101.00 ± 3.33	3.11 ± 0.16	103.67 ± 5.33	3.09 ± 0.21	103.00 ± 7.00
	1	3.06 ± 0.18	102.00 ± 6.00	3.16 ± 0.20	105.33 ± 6.67	3.17 ± 0.12	105.67 ± 4.00
	2	3.18 ± 0.15	106.00 ± 5.00	3.08 ± 0.15	102.67 ± 5.00	2.98 ± 0.25	99.33 ± 8.33
	3	2.92 ± 0.21	97.33 ± 7.00	3.10 ± 0.10	103.33 ± 3.33	N.A.	N.A.
	12	2.90 ± 0.19	96.67 ± 6.33	2.97 ± 0.21	99.00 ± 7.00	N.A.	N.A.
40	0	3.03 ± 0.10	101.00 ± 3.33	3.11 ± 0.16	103.67 ± 5.33	3.09 ± 0.21	103.00 ± 7.00
	1	3.08 ± 0.21	102.67 ± 7.00	3.18 ± 0.09	106.00 ± 3.00	3.08 ± 0.21	102.67 ± 7.00
	2	2.91 ± 0.19	97.00 ± 6.33	2.96 ± 0.11	98.67 ± 3.67	2.88 ± 0.09	96.00 ± 3.00
	3	2.94 ± 0.18	98.00 ± 6.00	2.98 ± 0.09	99.33 ± 3.00	N.A.	N.A.
	12	2.89 ± 0.22	96.33 ± 7.33	2.88 ± 0.17	96.00 ± 5.67	N.A.	N.A.

N.A., not available.

**Table 9:** Residual niaprazine concentration (mg·mL^−1^) and drug content (% of theoretical concentration, calculated relative to the nominal niaprazine concentration of 3 mg·mL^−1^) in formulations prepared using Puccini and Mozart sugar-free vehicles and stored for 12 months, and Wagner vehicle stored for 3 months, at 4–8 °C. Concentrations and standard deviations (SD) were determined by HPLC analysis using diode array detection (DAD, λ = 254 nm) and mass spectrometric detection (MS, TIC mode). Data represent the mean of three independent experiments.

Temperature (°C)	Analytical Technique	Niaprazine Concentration (mg·mL^−1^) and Drug Content (%) ± S.D.					
		Puccini		Mozart Sugar Free		Wagner ^a^	
4–8	HPLC-DAD	2.93 ± 0.06	96.67 ± 2.00	2.94 ± 0.02	98.00 ± 0.67	2.97 ± 0.09	99.00 ± 3.00
	HPLC-MS	2.94 ± 0.11	98.00 ± 3.67	2.92 ± 0.08	97.33 ± 2.67	3.23 ± 0.12	107.70 ± 3.88
25	HPLC-DAD	3.29 ± 0.15	109.67 ± 5.00	3.29 ± 0.03	109.67 ± 0.96	N.A.	N.A.
	HPLC-MS	2.99 ± 0.12	99.67 ± 4.00	3.24 ± 0.14	108.00 ± 4.67	N.A.	N.A.
40	HPLC-DAD	2.94 ± 0.10	98.00 ± 3.33	2.85 ± 0.06	95.00 ± 2.00	N.A.	N.A.
	HPLC-MS	2.92 ± 0.04	97.33 ± 1.33	2.89 ± 0.04	96.33 ± 1.33	N.A.	N.A.

^a.^ data obtained at 3 months; N.A., not available.

**Table 10:** Microbiological quality, pH stability and visual alteration of niaprazine syrup formulations (single batch), evaluated according to the European Pharmacopoeia (12th Edition) to 2 months stored at two different temperature (4–8 and 25 °C).

Formulation	Storage Condition (°C)	Time Point (Month)	TAMC (CFU·mL^−1^)	TYMC (CFU·mL^−1^)	*Escherichia coli*	pH Variation vs. t = 0	Visual Alteration
1	4–8	2	≤10^3^	≤10^2^	Absent	<0.5	None
	25	2	≤10^3^	≤10^2^	Absent	<0.5	None
2	4–8	2	≤10^3^	≤10^2^	Absent	<0.2	None
	25	2	≤10^3^	≤10^2^	Absent	<0.1	None
3	4–8	2	≤10^3^	≤10^2^	Absent	<0.1	None
	25	2	≤10^3^	≤10^2^	Absent	<0.1	None

## Data Availability

The original contributions presented in this study are included in the article. Further inquiries can be directed to the corresponding author.
